# Construction of a Prognostic Immune-Related LncRNA Risk Model for Gastric Cancer

**DOI:** 10.1155/2022/5137627

**Published:** 2022-06-25

**Authors:** Wei Ding, Pengcheng Sun, Yulin Tan, Huaji Jiang, Cheng Xi, Ling Zhuang, Yixin Xu, Xuezhong Xu

**Affiliations:** ^1^Department of General Surgery, Wujin Hospital Affiliated to Jiangsu University, Changzhou 213017, China; ^2^Department of General Surgery, The Wujin Clinical College of Xuzhou Medical University, Changzhou 213017, China; ^3^Department of Oncology, The Third Affiliated Hospital of Soochow University, Changzhou 213001, China

## Abstract

Gastric cancer (GC) is one of the most common malignancies, and novel prognostic biomarkers for it are urgently required. This study is aimed at screening a group of immune-related lncRNAs (IRLs) in predicting the prognosis of GC patients. Genetic and clinical information from the 360 GC patients was included in this study. Eight IRLs in lncRNA-miRNA-mRNA network were screened out according to differential expression analysis. A novel risk score model with three IRLs (MIR4435-1HG, UCA1, and RP11-617F23.1) were identified, and patients were assigned to a high-risk group and a low-risk group. Patients in the low-risk group had a better prognosis. In addition, two nomograms were developed to predict the prognosis of GC. We evaluated the correlation between IRLs and the immune infiltration level of GC using TIMER. Furthermore, we verified that RP11-617F23.1 was significantly upregulated in human GC tissues compared with their adjacent tissues. And, patients with high RP11-617F23.1 expression in tumor tissues had poorer survival. In conclusion, we established a novel risk model based on IRLs for predicting the prognosis of GC. Meanwhile, a novel IRL, RP11-617F23.1, could serve as a predictor of prognosis for patients with GC.

## 1. Introduction

Gastric cancer (GC) is one of the commonest malignancies all over the world, with nearly one million new cases each year, accounting for 5.7% of all malignant tumors [1]. The incidence and mortality rate of GC, respectively, rank the 5th and 3rd among malignant tumors, and the incidence in Asia ranks the first [2]. At present, radical resection is still the most efficient option for early GC patients with low risk of lymph node metastasis. However, most patients are in moderate and advanced stages when they are diagnosed, and some patients already have local or distant metastasis because the early GC is not obvious [3,4]. Most of them are intolerance of operation, and even if they could be excised surgically, they would be prone to relapse and metastasis, with a poor prognosis, and the 5-year survival rate only reaches 30% [5,6]. Therefore, the identification of key regulators and the elucidation of the potential mechanisms for initiating and promoting the occurrence and metastasis of GC are conducive to the formulation of a reasonable postoperative follow-up plan and the adoption of targeted interventions to improve the survival rate. It is urgently required to clarify the molecular mechanism of GC and to find ideal markers for early diagnosis and specific therapeutic targets.

As far back as 1909, Ehrlich demonstrated that the immune balance had the effect of inhibiting most tumors and played a significant role in preventing tumor progression [7]. The immune cells can specifically recognize antigens expressed on the tumor cell surface and generate immune responses via releasing cytokines to act directly on the tumor cells and inhibit tumor growth [8]. Although tumor-related immune cells within the tumor microenvironment (TME) play a role in eliminating tumor cells in the anti-tumor process, some tumor cells still escape under immune surveillance [9]. It is increasingly recognized that the TME has an important role in tumor progression [10]. These tumor-associated immune cells may have antitumor or protumor effects. Immune escape as a new marker of cancer provides opportunities for new strategies for cancer treatment. As RNA sequencing developed, novel therapeutic biomarkers at the gene level in the TME were unearthed in abundance [11].

Long noncoding RNA (lncRNA) is a kind of RNA with a structure of more than 200 nucleotides and no functional open reading frame. Research showed that lncRNA was involved in various biological functions, including the regulation of growth, aging, differentiation, pyroptosis, apoptosis, and tumorigenesis [12]. Various lncRNAs have been found to affect tumor growth and invasion and immune response. For example, lncRNA SATB2-AS1 has been clarified to inhibit tumor metastasis and affect the TME in colorectal cancer by targeting SATB2 [13]. Mesenchymal stem cells can induce liver cancer through lncRNA-MUF interaction with miR-34a and ANXA2 [14]. LncRNA SNHG1 has been demonstrated to regulate the differentiation of regulatory T cells, thus affecting immune escape of breast cancer by targeting miR-448/IDO [15]. LncRNAs can regulate TME and have significant role in immunotherapy. However, research on immune-related lncRNA (IRL) in GC has been relatively sparse.

The purpose of our research was to screen novel immune-related lncRNAs, which might serve as predictors and therapeutic targets in GC. We performed differential expression analysis, univariate and multivariate Cox regression analysis, Kaplan–Meier survival analysis, TIMER database analysis, and other analysis to identify IRLs and evaluate the predictive ability and therapeutic potential.

## 2. Methods

This study was approved by the Ethics Review Committee of Wujin Hospital affiliated with Jiangsu University (no. 202121).

### 2.1. Data Acquisition and Preprocessing

GPL16956 Agilent-045997 Arraystar human lncRNA microarray V3 (Probe Name Version) platform was used to obtain the microarray dataset GSE122530 which was pretreated and standardized from the Gene Expression Omnibus (GEO) repository [16]. There were six paired GC and normal tissue samples. The expression of RNA sequencing and clinical data associated with GC were collected from The Cancer Genome Atlas (TCGA) [17], which included 354 GC and 41 paracancer tissue samples. Clinical information in TCGA was collected, including age, gender, tumor stage, and differentiated degree.

### 2.2. Data Annotation

Download the human reference genome sequence file (GRCh38.p2.genome.fa) from the GENCODE database [18]. Seqmap software was applied to match all probe sequences to the reference genome [19]. We kept the unique mapped reads and obtained the corresponding genes of each probe. We annotate these probes according to GENCODE by using the information of the probes on chromosomes. Finally, the probes were paired with Gene Symbols, and the unpaired probes were removed.

### 2.3. Differential Expression Analysis

After obtaining the gene expression matrix through the previous gene annotation, Limma package [20] was used to obtain the adjusted *P* value and |logFC| by empirical Bayes and linear regression along with Benjamini and Hochberg multiple comparison methods. Differentially expressed mRNAs and lncRNAs were identified, while adjusted *P* value <0.05 and |logFC| >0.585; differentially expressed miRNAs were identified, while adjusted *P* value <0.05 and |logFC| >1. After the above difference analysis, we select the intersection of differentially expressed mRNA and lncRNA in the two groups according to upregulation and downregulation to explore the common differentially expressed lncRNAs and mRNAs.

### 2.4. Building the ceRNA Network

The lncRNA-miRNA-mRNA network was built according to the ceRNA hypothesis [21]. In the miRNA module of miRWalk 3.0, input the miRNA list, set the species to “human,” set the score value >0.85, and run to obtain the predicted miRNA-mRNA regulatory relationship pairs, which also appeared in TargetScan [22], miRDB [23], and miRTarBase [24] databases. The miRNA-related lncRNAs were predicted using Prediction Module of DIANA-LncBase Predicted v.2 database, and the regulation relationship of score greater than 0.6 was selected. According to the common differentially expressed miRNAs and miRNA-mRNA and lncRNA-miRNA regulatory relationship pairs obtained above, we built the lncRNA-miRNA-mRNA network.

### 2.5. Immune-Related ceRNA Network

Download the immune genes in the Immunology Database and Analysis Portal (ImmPort) from the InnateDB database [25] and match them with the ceRNA network to obtain the immune-related ceRNA network. The lncRNAs in the immune-related ceRNA network were identified as IRLs.

### 2.6. Univariate and Multivariate Cox Regression and Kaplan–Meier Survival Analysis

Preprocessing of survival data: to ensure the accuracy of survival time, samples with a survival status of 0 (survival) and survival time <1 month were considered a failure of follow-up in this analysis, and these samples were removed from the total samples. Finally, 339 samples were retained for overall survival (OS) data and 266 samples for disease-free survival (DFS) data. Univariate analyses from the survival package (version 3.2-7) were performed with Cox regression analysis for IRLs. After univariate Cox analysis, lncRNAs with *P* value <0.05 were screened out.

A novel risk model was developed for predicting the prognosis. The risk score (RS) was calculated as follows:

(1) $$\begin{array}{c} \text{RS}=\beta_{\text{gene}1}\times {\text{Expr}}_{\text{gene}1}+\beta_{\text{gene}2}\times {\text{Expr}}_{\text{gene}2}+\ldots +\beta_{\text{genen}}\times {\text{Expr}}_{\text{genen}}, \end{array}$$

where *β*_gene_ indicated the regression coefficient *β* for each gene and Expr_gene_ indicated the expression value of the corresponding gene for each sample.

The appellate formula was used to calculate the RS of each sample. The optimal cut-off RS point was determined using maximally selected rank statistics according to the risk model. Two groups (low-risk and high-risk groups) of patients were divided according to the optimal cut-off RS point. The two groups were used to compared by Kaplan–Meier survival analysis.

### 2.7. Nomogram Model Construction

Univariate Cox regression analyses were used to sift out risk factors, based on RS, age, gender, tumor stage, and differentiated degree. Multivariate analyses were used to screen out independent risk factors with *P* < 0.05. The nomograms were constructed by using the rms package (version 6.1-0) with factors obtained above.

### 2.8. TIMER Database Analysis

We analyzed the expression of IRLs obtained above in different types of cancer and the correlation with the degree of immune infiltrates, including B cells, CD8+ T cells, CD4+ T cells, macrophages, neutrophils, and dendritic cells, via TIMER database [26].

### 2.9. Patients and Samples

The present study included 64 patients with gastric cancer. All patients underwent radical open gastrectomy in Wujin Hospital from January 2014 to October 2014. The inclusion criteria were as follows: [1] had detailed history, examination, and laboratory investigations; [2] did not have distant metastases; [3] no antitumor therapy was performed before surgery; and [4] complete follow-up data were available. The adjacent normal tissues were also collected 3–5 cm away from the edge of the tumor.

### 2.10. Quantitative Real-Time Polymerase Chain Reaction

Total RNA was extracted using Trizol® reagent (Shanghai Pufei Biotech Co., Ltd.) based on the supplier's instruction. M-MLV kit (Promega Biotech Co., Ltd) was used to obtained cDNA by reverse transcription. qPCR was conducted using the SYBr Master Mix (Takara Biomedical Technology Co., Ltd.) and the Real‐Time PCR System (LightCycler 480 II) in the 12 *μ*l reaction mixture with the following conditions: initial denaturation at 95°C for 30 sec, followed by 40 cycles of 95°C for 5 sec, 60°C for 30 sec, then followed by one cycle of 95°C for 15 sec, 60°C for 30 sec, and 95°C for 15 sec. The following primer information was used for qPCR: ACTB forward, 5′-GCGTGACATTAAGGAGAAGC-3′ and reverse, 5′-CCACGTCACACTTCATGATGG-3′; RP11-617F23.1 forward, 5′-ACCGCAGGCACTTGTGAAGA-3′ and reverse, 5′-AAGGGACATGCAGAGGGGAG-3′. For quantification of RNA levels, the ΔΔCT method was applied, and the internal reference gene ACTB was used for normalization.

### 2.11. Statistical Analysis

Group differences for continuous variables were analyzed by *t*-test or one-factor analysis of variance (one-way ANOVA). Group differences in the distribution of categorical variables were analyzed by the chi-square test. Survival analysis was conducted by log-rank tests. Survival curves were drawn using the Kaplan–Meier method. All statistical analyses were calculated with Prism 9.0 (GraphPad Software, LLC).

## 3. Results

### 3.1. Differential Analysis of Genes

**Figure 1:**
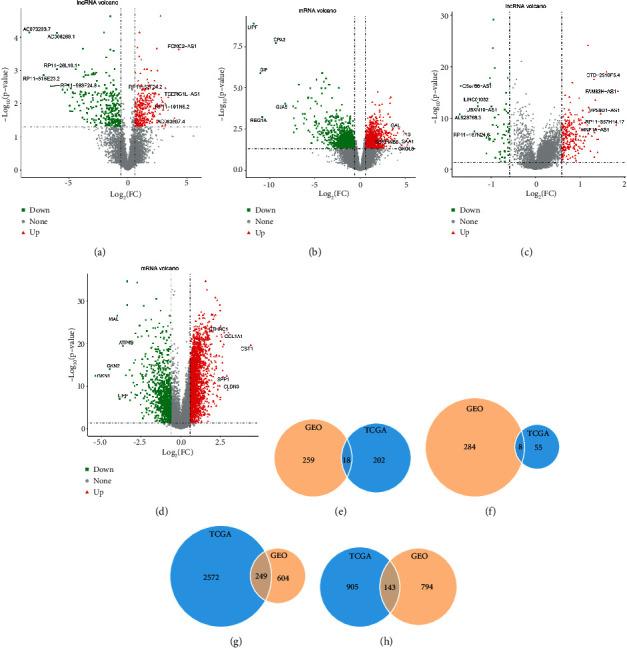
Volcano map and Venn diagram of differential genes. The volcano map of differential (a) lncRNA and (b) mRNA in GSE122530; the volcano map of differential (c) lncRNA and (d) mRNA in TCGA; (e) upregulated and (f) downregulated lncRNAs; and (g) upregulated and (h) downregulated mRNAs.

According to the differential analysis method described in the method, the results are shown in Table 1. The volcano map of the differential genes is shown in Figures 1(a)–1(d). After intersection analysis, a total of 392 common differential mRNAs and 26 common differential lncRNAs were achieved, as shown in Figures 1(e)–1(h).

### 3.2. Construction of Immune-Related ceRNA Network

**Figure 2:**
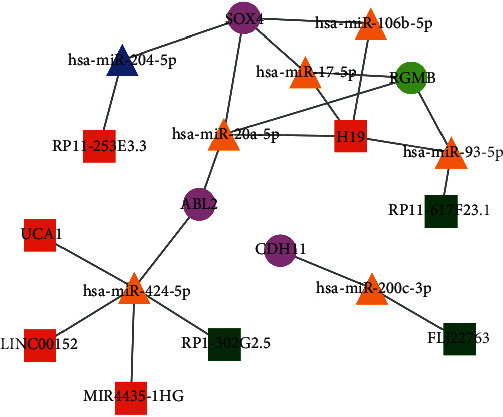
Immune-related lncRNA-miRNA-mRNA network. The pink circle stands for upregulated mRNA, the green circle stands for downregulated mRNA, the yellow triangle stands for upregulated miRNA, the blue triangle stands for downregulated miRNA, the red square stands for upregulated lncRNA, and the dark green square stands for downregulated lncRNA.

The target gene prediction tool miRWalk3.0 was used to predict the common differential mRNAs associated with differential miRNAs, and a total of 29 pairs miRNA-mRNA were obtained, including 14 miRNAs and 17 mRNAs. Furthermore, according to LncBase Predicted v.2 database, 12 lncRNAs were predicted associated with differential miRNAs. Based on the obtained lncRNA-miRNA and miRNA-mRNA relationship pairs, Cytoscape was used to construct the ceRNA network. Finally, 13 miRNAs, 12 lncRNAs, and 16 mRNAs were obtained, with a total of 58 regulatory pairs. With InnateDB database matching, we obtained 4 immune-related mRNAs (CDH11, RGMB, SOX4, and ABL2). By matching the above network, an immune-related ceRNA network was built, including 8 lncRNAs, 7 miRNAs, and 4 mRNAs, with a total of 21 regulatory pairs (Figure 2). These 8 lncRNAs were identified as immune-related lncRNAs.

### 3.3. Development of the OS and RFS Nomograms

**Figure 3:**
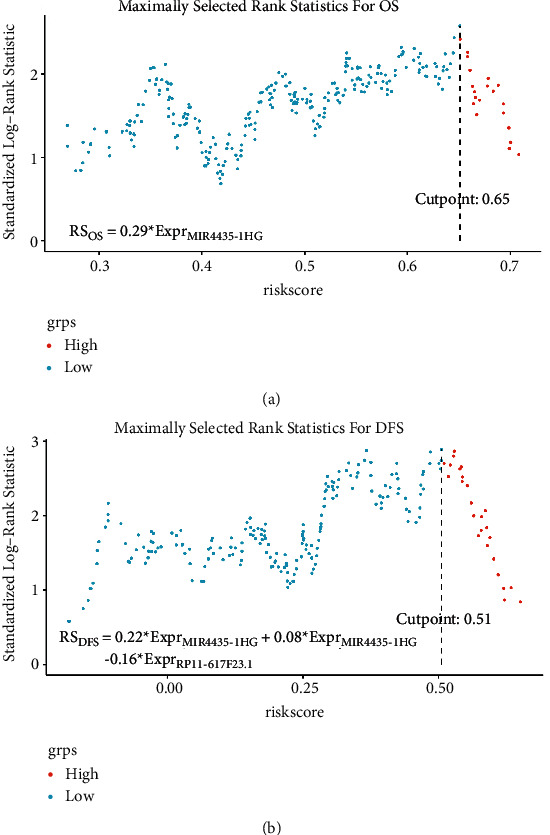
Maximally selected rank statistics. (a) Optimal cut-off value of RS for OS; (b) optimal cut-off value of RS for DFS (OS = overall survival; DFS = disease‐free survival; and RS = risk score).

**Figure 4:**
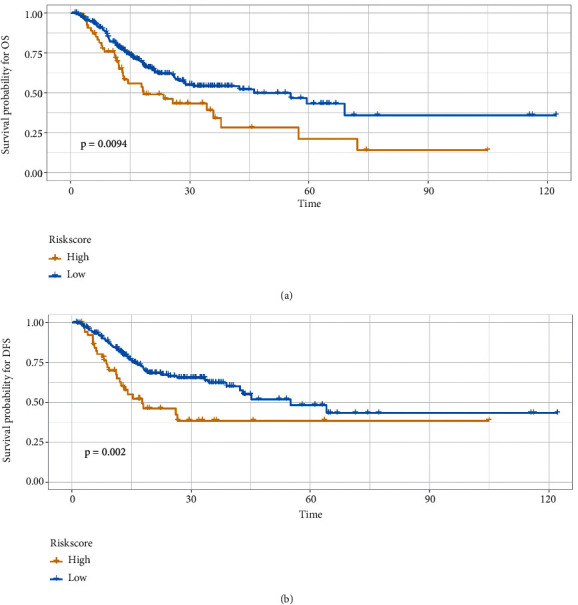
Survival analysis. (a) The Kaplan–Meier survival curves of OS according to RS. (b) The Kaplan–Meier survival curves of DFS according to RS (OS = overall survival; DFS = disease‐free survival; and RS = risk score).

One lncRNA associated with overall survival and three lncRNAs associated with disease-free survival were identified using univariate Cox analysis, and the results are shown in Table 2. The regression coefficient *β* was used to calculate the RS of each sample. The optimal cutoff RS points are shown in Figure 3. The patients were separated into a high-risk group and a low-risk group with the cut-off value. By survival analysis, the patients in low-risk group had significantly better OS and DFS as shown in Figure 4.

The RS was combined with clinical characteristics for univariate and multivariate regression analyses. Multivariate analysis showed that tumor stage (*P* < 0.01) and age (*P*=0.01) were closely related to OS (Table 3), while tumor stage (*P*=0.02) and RS (*P* < 0.01) were closely related to DFS (Table 4).

**Figure 5:**
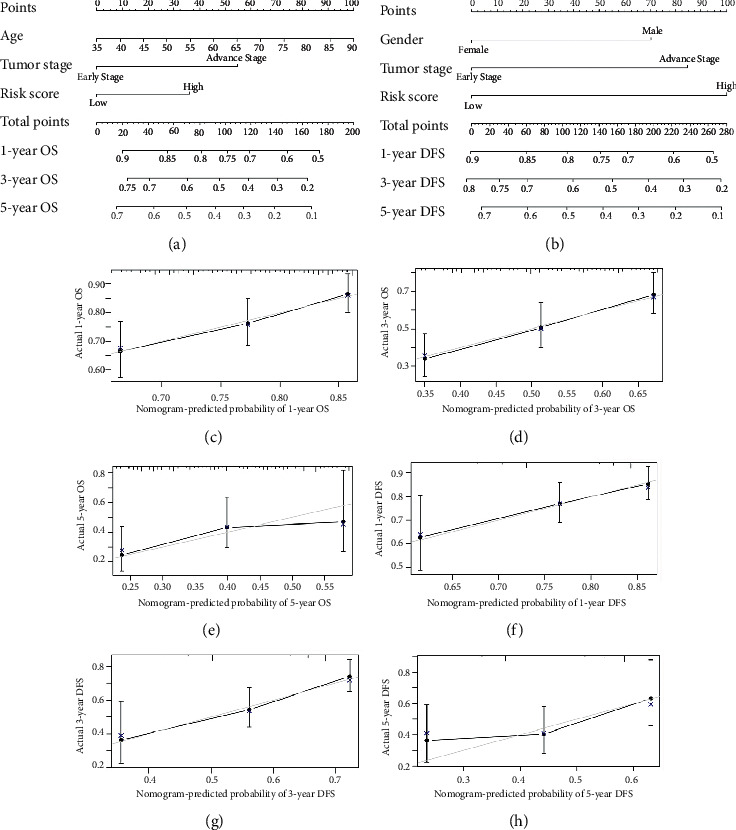
Nomograms for patients with gastric cancer. (a) The 1-, 3-, and 5-year overall survival nomogram. (b) The 1-, 3-, and 5-year disease-free survival nomogram. The calibration curves for predicting the (c–e) 1-, 3-, and 5-year overall survival and (f–h) 1-, 3-, and 5-year disease-free survival rates by nomogram prediction and actual observation in patients with gastric cancer.

The multivariate analyses identified that tumor stage, RS, and age were independent risk factors for GC patients. To better predict the prognosis at 1-, 3-, and 5-year OS and DFS of GC patients, we constructed nomograms of the variables above (Figures 5(a) and 5(b)). The calibration plot for the probability of OS and DFS had an optimal agreement between the two nomograms for probabilities and actual observation, respectively (Figures 5(c)–5(h)).

### 3.4. TIMER Database Analysis

**Figure 6:**
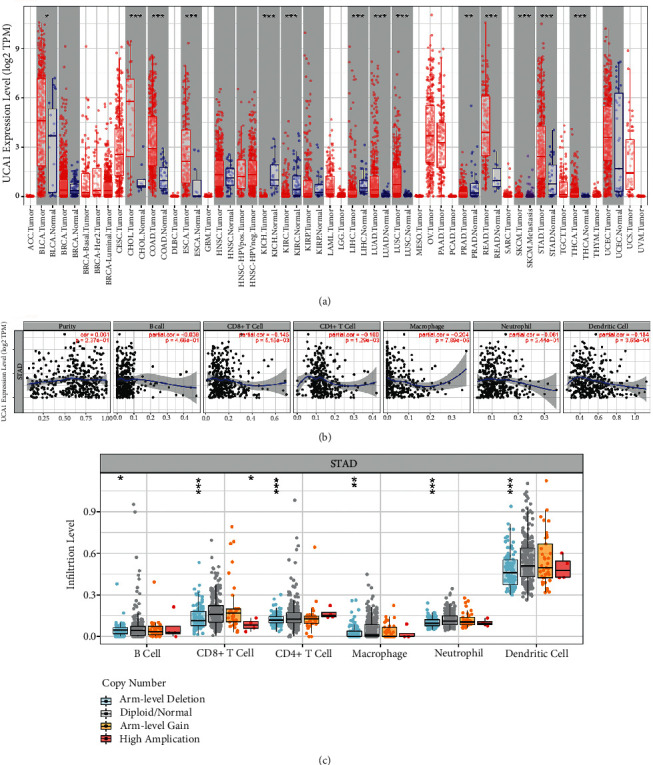
The association between UCA1 and the infiltration degree of immune cells. (a) Human UCA1 expression levels in different tumor types from TCGA database were determined by TIMER. (b) Correlation of UCA1 expression with the immune infiltration level in stomach adenocarcinoma. (c) Copy number distribution box plot of UCA1 in different immune cells in stomach adenocarcinoma (^*∗*^*P* < 0.05, ^*∗∗*^*P* < 0.01, and ^*∗∗∗*^*P* < 0.001).

The correlation between lncRNAs (MIR4435-1HG, UCA1, and RP11-617F23.1) and the infiltration degree of immune cells was described using TIMER database. However, only UCA1 was recorded in TIMER database. The expression levels of UCA1 in normal and primary tumor samples in all TCGA tumors are shown in Figure 6(a). The expression level of UCA1 was significantly higher in bladder urothelial carcinoma (BLCA), cholangiocarcinoma (CHOL), colon adenocarcinoma (COAD), esophageal carcinoma (ESCA), lung adenocarcinoma (LUAD), lung squamous cell carcinoma (LUSC), rectum adenocarcinoma (READ), stomach adenocarcinoma (STAD), and thyroid carcinoma (THCA) compared with adjacent normal tissues. However, UCA1 expression was significantly lower in kidney chromophobe (KICH), kidney renal clear cell carcinoma (KIRC), liver hepatocellular carcinoma (LIHC), and prostate adenocarcinoma (PRAD) compared with adjacent normal tissues. Then, we assessed the association between the immune infiltration level and the UCA1 expression level in stomach adenocarcinoma using TIMER. The results showed that the expression level of UCA1 was closely related to the infiltrating levels of CD8+ T cells, CD4+ T cells, macrophages, and dendritic cells, as shown in Figure 6(b). And, UCA1 had the highest copy number in DC cells in stomach adenocarcinoma, as shown in Figure 6(c).

### 3.5. The Verification of Clinical Role of RP11-617F23.1 in Gastric Cancer

**Figure 7:**
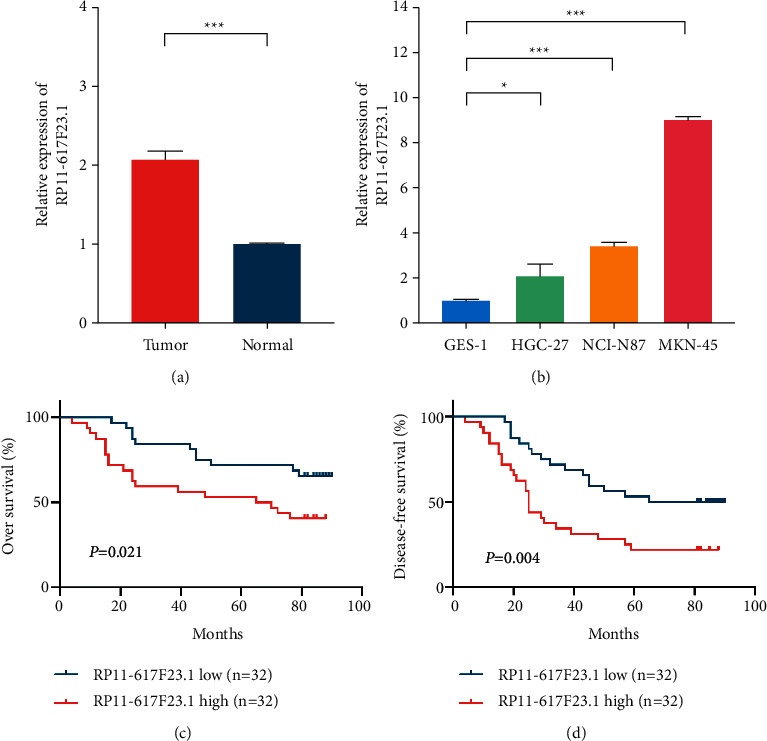
The verification of clinical role of RP11-617F23.1 in gastric cancer. (a) RP11-617F23.1 was significantly upregulated in human GC tissues (*n* = 64) compared with their corresponding adjacent tissues (*n* = 64). (b) RP11-617F23.1 was upregulated in gastric cancer cell lines compared with gastric epithelial cell. (c, d) Patients with high RP11-617F23.1 expression in tumor tissues had poorer overall survival (OS) and disease‐free survival (DFS) than patients with low RP11-617F23.1 expression (^*∗*^*P* < 0.05, ^*∗∗∗*^*P* < 0.001).

Based on the three prognostic related IRLs (MIR4435-1HG, UCA1, and RP11-617F23.1) obtained above were analyzed further. As the function of MIR4435-1HG and UCA1 has been verified before [27,28], we only tested the effect of RP11-617F23.1. First, we detected the relative expression levels of RP11-617F23.1 in GC tissues and adjacent tissues, as well as four cell lines (GES-1, NCI-N87, MKN-45, and HGC-27). As shown in Figure 7(a), RP11-617F23.1 was significantly upregulated in human GC tissues (*n* = 64) compared with their corresponding adjacent tissues (*n* = 64). Similarly, it was significantly upregulated in gastric cancer cell line (NCI-N87, MKN-45, and HGC-27) compared with gastric mucosa cell (GES-1), as shown in Figure 7(b). To further validate its clinical effect, we compared it with clinical features and survival data. Patients were separated into two groups (high and low) based on the median of the relative expression of RP11-617F23.1 in tumor tissues. As shown in Table 5, patients with higher expression of RP11-617F23.1 in tumor tissues had higher NRL value (*P* < 0.001), more advanced T stage (*P*=0.004) and poorer tumor differentiation (*P*=0.039) and a higher probability of lymph node metastasis (*P*=0.044). Additionally, the prognostic value of RP11-617F23.1 for patients with GC was assessed by Kaplan–Meier survival analysis. The results identified that patients with high RP11-617F23.1 expression in tumor tissues had poorer OS and DFS than patients with low RP11-617F23.1 expression (OS : *P*=0.021, Figure 7(c); DFS : *P*=0.004, Figure 7(d)). Thus, our data suggested that RP11-617F23.1 was a novel marker indicating poor GC prognosis.

## 4. Discussion

In the current study, a total of 360 GC and 47 pericarcinomatous tissues derived from two datasets (TCGA and GSE122530) were incorporated into the calculation of the differential expression lncRNAs in patients with GC. Overall, 4723 immune-related mRNAs were downloaded from the InnateDB. Three IRLs (MIR4435-1HG, UCA1, and RP11-617F23.1) were confirmed to be significantly associated with the prognosis of GC. GC patients could be separated into two groups by the risk model based on the three IRLs. The regression analyses of clinical information and RS were performed to identify the independent prognostic factors (tumor stage, age, and RS). The nomograms were constructed based on tumor stage, age, and RS to predict OS and DFS for GC patients visually. Then, the calibration plot identified that the two nomograms had high prediction accuracy. We also found that UCA1 expression was significantly associated with various immune cells, while the other two IRLs were not recorded in the TIMER database.

In our study, MIR4435-1HG and UCA1 were identified to exhibit cancer-promoting activity, while the clinical effect of RP11-617F23.1 was contradictory. Both UCA1 and MIR4435-1HG had been previously reported in GC. In the previous study, the expression of serum UCA1 was suggested to be closely associated with the differentiation of cancer cells in GC [29]. Additionally, UCA1 could promote cell proliferation and invasion of GC by regulating the miR-590-3p/CREB1 signal pathway [30]. The upregulation of UCA1 could promote the invasion and migration in GC [31]. Recent research has shown that UCA1 would act as an antitumor miRNA inhibitor to facilitate proliferation, migration, and immune escape and inhibit apoptosis in GC [28]. And, UCA1 could increase resistance to cisplatin in GC via recruiting EZH2 and activating the PI3K/AKT pathway [32]. MIR4435-1HG was also known as LINC00978, MIR4435-2HG, and AWPPH. In the previous study, the expression level of LINC00978 has been suggested to be significantly related to tumor size, lymphatic metastasis, and tumor stage [33]. Additionally, lncRNA LINC00978 could contribute to tumor development by regulating the microRNA-497/NTRK3 axis in GC [27]. However, the upregulation of lncRNA AWPPH was demonstrated to inhibit the proliferation and invasion of gastric cancer cells via the miR-203a/DKK2 axis in another study [34]. Recent study has shown that MIR4435-2HG could promote tumor metastasis in GC via targeting Wnt/*β*-catenin and desmoplakin. By contrast, there no reports concerning RP11-617F23.1 in GC so far. RP11-617F23.1 was also known as ZNF710-AS1-201 according to Ensembl database. Only one report concerning ZNF710-AS1-201, which demonstrated that the overexpression of ZNF710-AS1-201 was correlated with poor prognosis for patients with clear cell renal cell carcinoma [35]. This conclusion was consistent with our verification result.

Recently, a few studies of the influence of lncRNAs on the tumor immune microenvironment of GC have been reported. HOTAIR was found to upregulate COL5A1, which was correlated with immune infiltration and promote the growth and metastasis of GC by sponging miR-1277-5p [36]. LINC00941 was correlated with the immune environment in GC [37]. LINC00963 promoted the development of GC by targeting miR-612/CDC5L axis and mediated dendritic cell-related antitumor immune response [38]. CXXC finger protein 4 inhibited the immune escape of GC cells by acting on the ELK1/MIR100HG pathway [39]. MALAT1, as a sponge for miR-125a, regulates IL-21R signaling, participates in immune regulation of immune cells and tumor progression, and is a risk factor for survival and recurrence in GC [40]. Interestingly, RP11-617F23.1 was closely related to neutrophil-to-lymphocyte ratio (NLR) in the present study, which reflected the immune status of patients. In addition, previous studies have identified that NRL was also significantly associated with the prognosis of GC [41]. Therefore, the molecular mechanism of RP11-617F23.1 as an IRL deserves further study, including immunoassay and immune escape.

In contrast to the previous research on IRLs in GC [42–44], the current study comprehensively evaluated the immune-related ceRNA network for the first time, established the risk model, and preliminarily validated the related lncRNAs. Additionally, the finding also suggested that RP11-617F23.1 may be used as a prognostic predictor for GC. However, the present study still had a few limitations. First, the sample size of survival analysis was not large enough. And, the detailed molecular mechanisms of RP11-617F23.1 require further investigation.

## 5. Conclusion

We established a novel risk model based on IRLs for predicting the prognosis of GC. Meanwhile, a novel IRL, RP11-617F23.1, could act as a predictor of prognosis for patients with GC. This provided a theoretical basis for tumor prevention and immunotherapy.

## Figures and Tables

**Table 1:** The number of differential genes.

	GSE122530			TCGA		
	Up	Down	Total	Up	Down	Total
mRNA	852	936	1788	2821	1048	3869
LncRNA	277	292	569	219	62	281
miRNA	—	—	—	71	10	81

**Table 2:** Univariate analysis of overall survival and disease-free survival.

	Overall survival			Disease-free survival		
	HR	95% CI	*P* value	HR	95% CI	*P* value
MIR4435-1HG	1.34	[1.03–1.76]	0.03^*∗*^	1.44	[1.04–1.97]	0.03^*∗*^
H19	1.06	[0.98–1.14]	0.15	1.04	[0.96–1.14]	0.34
RP11-617F23.1	0.87	[0.71–1.06]	0.16	0.77	[0.59–0.99]	0.04^*∗*^
FLJ22763	0.83	[0.61–1.14]	0.26	0.84	[0.58–1.22]	0.36
RP11-253E3.3	0.9	[0.63–1.28]	0.55	0.9	[0.57–1.41]	0.64
UCA1	1.02	[0.94–1.12]	0.58	1.11	[1.01–1.23]	0.03^*∗*^
LINC00152	1.07	[0.83–1.37]	0.61	1.14	[0.85–1.54]	0.38
RP1-302G2.5	1.01	[0.74–1.38]	0.94	1.08	[0.76–1.54]	0.67

Note: overall survival, *β*_MIR4435-1HG_ = 0.29; disease-free survival, *β*_MIR4435-1HG_ = 0.22, *β*_UCA1_ = 0.08, and *β*_RP11-617F23.1_ = −0.16; ^*∗*^statistically significant.

**Table 3:** Univariate and multivariate analysis of overall survival.

	Univariate analysis			Multivariate analysis		
	HR	95% CI	*P* value	HR	95% CI	*P* value
Tumor stage (early vs. advance)	0.553	[0.382–0.800]	0.002^*∗*^	0.511	[0.355–0.736]	<0.001^*∗*^
Risk score (low vs. high)	0.654	[0.434–0.985]	0.042^*∗*^	0.642	[0.428–0.964]	0.033^*∗*^
Age (years)	1.026	[1.008–1.044]	0.004^*∗*^	1.023	[1.005–1.040]	0.011^*∗*^
Differentiated degree (low vs. high)	0.744	[0.511–1.083]	0.122	—	—	—
Gender (female vs. male)	1.211	[0.830–1.768]	0.321	—	—	—

Note: HR = hazard ratio; CI = confidence interval; ^*∗*^statistically significant.

**Table 4:** Univariate and multivariate analysis of disease-free survival.

	Univariate analysis			Multivariate analysis		
	HR	Upper 95	*P* value	HR	Upper 95	*P* value
Tumor stage (early vs. advance)	0.477	[0.298–0.763]	0.010^*∗*^	0.549	[0.357–0.843]	0.006^*∗*^
Risk score (low vs. high)	0.563	[0.364–0.871]	0.002^*∗*^	0.492	[0.308–0.785]	0.003^*∗*^
Age (years)	1.671	[1.025–2.723]	0.040^*∗*^	1.647	[1.014–2.674]	0.044^*∗*^
Differentiated degree (low vs. high)	0.851	[0.548–1.322]	0.472	—	—	—
Gender (female vs. male)	1.001	[0.981–1.021]	0.945	—	—	—

Note: HR = hazard ratio; CI = confidence interval; ^*∗*^statistically significant.

**Table 5:** Comparation of the clinicopathological characteristics between low and high expression groups.

Variables	RP11-617F23.1		*P* value
	Low expression (*n* = 32)	High expression (*n* = 32)	
Age, years	65.94 ± 6.90	67.84 ± 8.49	0.329
Sex, male	17 (53.1%)	22 (68.8%)	0.200
CEA, U/ml	20.54 ± 72.16	7.31 ± 20.63	0.692^#^
NLR	2.00 ± 0.76	3.81 ± 2.34	<0.001^#*∗*^
Tumor differentiation, poor	16 (50.0%)	24 (75.0%)	0.039^*∗*^
T stage, III-IV	15 (46.9%)	26 (81.3%)	0.004^*∗*^
Lymphatic invasion, positive	14 (43.8%)	22 (68.8%)	0.044^*∗*^

Note: mean ± standard deviation, number (percent); NLR: neutrophil lymphocyte ratio; ^#^Mann–Whitney test; ^*∗*^statistically significant.

## Data Availability

The RNA sequencing profiles can be obtained from The Cancer Genome Atlas (TCGA) (https://xenabrowser.net/) and Gene Expression Omnibus (GEO) (https://www.ncbi.nlm.nih.gov/geo/). The immune-related genes can be downloaded from the InnateDB database (https://www.innatedb.ca/index.jsp). The datasets used and/or analyzed during the current study are available from the corresponding author on reasonable request.
